# Bioinformatics analysis of capsid protein of different subtypes rabbit hemorrhagic disease virus

**DOI:** 10.1186/s12917-019-2161-9

**Published:** 2019-11-27

**Authors:** Ruibin Qi, Jie Zhu, Qiuhong Miao, Aoxing Tang, Dandan Dong, Xiaoxue Wang, Guangqing Liu

**Affiliations:** Innovation Team of Small animal Infectious Disease, Shanghai Veterinary Research Institute, Chinese Academy of Agricultural Sciences (CAAS), Shanghai, 200241 People’s Republic of China

**Keywords:** RHDV, RHDVa, RHDVb, Bioinformatics analysis

## Abstract

**Background:**

Rabbit Hemorrhagic Disease Virus (RHDV) belongs to the *Caliciviridae* family, is a highly lethal pathogen to rabbits. Increasing numbers of studies have demonstrated the existence of antigenic variation in RHDV, leading to the emergence of a new RHDV isolate (RHDVb). However, the underlying factors determining the emergence of the new RHDV and its unpredictable epidemiology remain unclear. To investigate these issues, we selected more than 184 partial and/or complete genome sequences of RHDV from GenBank and analyzed their phylogenetic relationships, divergence, and predicted protein modification sites.

**Results:**

Phylogenetic analysis showed that classic RHDV isolates, RHDVa, and RHDVb formed different clades. It’s interesting to note that RHDVa being more closely related to classic RHDV than RHDVb, while RHDVb had a closer genetic relationship to Rabbit Calicivirus (RCV) than to classic RHDV isolates. Moreover, divergence analysis suggested that the accumulation of amino acid (aa) changes might be a consequence of adaptive diversification of capsid protein (VP60) during the division between classical RHDV, RHDVa, RHDVb, and RCV. Notably, the prediction of N-glycosylation sites suggested that RHDVb subtypes had two unique N-glycosylation sites (aa 301, 362) but lacked three other N-glycosylation sites (aa 45, 308, 474) displayed in classic RHDV and RHDVa VP60 implying this divergence of N-glycosylation sites in RHDV might affect viral virulence. Analysis of phosphorylation sites also indicated that some phosphorylation sites in RHDVa and RHDVb differed from those in classic RHDV, potentially related to antigenic variation in RHDV.

**Conclusion:**

The genetic relationship between RHDVb and RCV was closer than classic RHDV isolates. Moreover, compared to RHDV and RHDVa, RHDVb had two unique N-glycosylation sites but lacked three sites, which might affect the virulence of RHDV. These results may provide new clues for further investigations of the origin of new types of RHDV and the mechanisms of genetic variation in RHDV.

## Background

Rabbit hemorrhagic disease (RHD) is a highly fatal infectious disease caused by RHDV, which is first discovered in China in 1984. And it has been subsequently spread worldwidely within a few years, resulting in great economic losses in the rabbit industries [1, 2]. RHDV, European Brown Hare Syndrome Virus (EBHSV) as well as the non-pathogenic rabbit calicivirus (RCV) both belong to the genus *Lagovirus,* family *Caliciviridae* [3–5]. RHDV isolates have been classified into three subtypes including classic RHDV (G1-G5), RHDVa (G6), and RHDVb (G1.2) [6–8]. The new RHDV variant, called RHDV2/b, was identified for the first time in 2011. The RHDVb infection spectrum is expanded, including not only the European rabbit (*Oryctolagus cuniculus*) [9] but also the Sardinian cape hare (*L. capensis*) [10], the Corsican hare (*L. corsicanus*) [9], and the European hare (*L. europaeus*) [11]. RHDVb also cause the death of young rabbits aged 2–3 weeks or rabbit vaccinated, which suggests that classic RHDV and RHDVb may use different receptors [7]. Now RHDVb has been reported in many countries in Europe, Australia, Africa, and North America [7–9, 12–19] and has replaced classic RHDV as the major cause of RHD in many areas [20–22].

RHDV contains a 7437-nucleotide positive-sense single-stranded genomic RNA, which is composed of two slightly overlapping open reading frames (ORFs) and a 2.2-kb designated subgenomic RNA. ORF1 encodes a large polyprotein cleaved by a virus-encoded protease into the main capsid protein of RHDV VP60 and seven mature non-structural proteins (p16, p23, helicase, p29, Vpg, protease, RdRp) [23–26]. And ORF2 encodes another structural protein (VP10) [24] playing part in the replication and release from infected host cells of RHDV [27].

VP60, the major structural protein of RHDV, determining the differences of three subtypes of RHDV in genetic, antigenic, and epidemiological diversity and immunological response, consists of three domains: the N-terminal arm (NTA, aa 1–65), the shell (S, aa 66–229), the protrusion (P, aa 238–579), and a short hinge (aa 230–237) that connects the S and P domains [28, 29]. The P domain is further divided into P1 (aa 238–286, 450–466, and 484–579) and P2 (aa 287–449 and 467–483) sub-domains located at the most exposed region of VP60. P2 subdomain shows the greatest genetic variation [30] and plays important role in binding to histo-blood group antigens (HBGAs) or host tissues [31, 32].

In this study, we attempt to explain the relations among classic RHDV, RHDVa, RHDVb and RCV, which will establish a foundation to reveal the emergence and epidemiology differences between RHDV and RHDVb.

## Results

### Phylogenetic analysis of RHDV

**Fig. 1:**
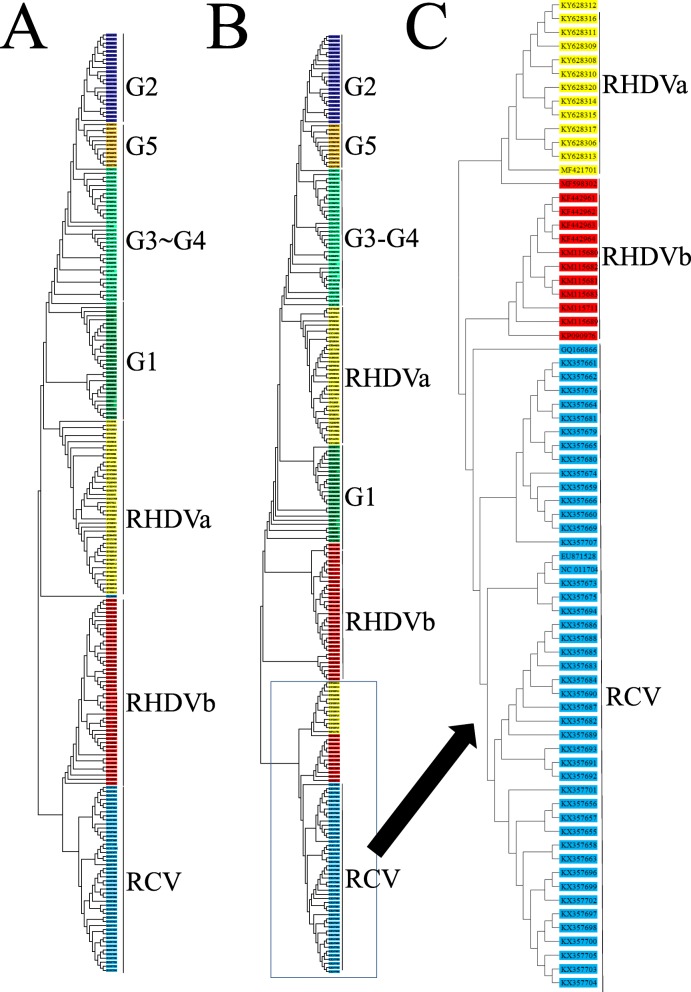
Phylogenetic analysis of RHDV isolates. Maximum likelihood tree for RHDV using MEGA5 with the sequence of VP60 (**a**), and complete sequence (**b**). Numbers on branches represent bootstrap values (based on 1000 replications)

Phylogenetic analysis of RHDV isolates based on VP60 showed that RHDVb was more closely related to RCV than RHDV (Fig. 1a), but another phylogenetic tree based on the complete sequence showed some RHDVa and RHDVb isolates were more closely related to RCV than to RHDV (Fig. 1b). The amino acid alignment showed that amino acids which deficient in 136,137 and 716 among these RHDVa and RHDVb isolates were the same as in the RCV isolates. Those isolates may have a recombinant history and thus be transitions from RCV to RHDV [33, 34]. We used the phylogenetic tree based on VP60 to classify the subtypes of RHDV (Fig. 1a). The classic RHDV, RHDVa, and RHDVb subtypes and RCV sequences consisted of 95, 43, 46 and 46 isolates, respectively.

### Adaptive diversification among classic RHDV, RHDVa, and RHDVb

In theory, the variations of a sequence were supposed to have a similar evolutionary process for they are linked tightly [35]. Nevertheless, recombination among the parts of different sequences would obscure the evolutionary process, which has been found to exist universally in different subtypes of RHDV [36, 37]. Aiming to determine the potential adaptive variations which are associating with the split of the three phylogenic subtypes (classic RHDV, RHDVa, and RHDVb) from a common ancestor, we firstly take the RDP v4.56 package to detect the recombination covering the entire coding sequences of the 184 RHDV genomes and identified eight putative recombinant isolates (MF598302, MF421679, KP129396, KY628317, KY765609, KY628317, EF558585, EF558586), whose the donors belonged to different subtypes (Additional file 2: Table S2). In addition, these sequences were validated by BOOTSCAN, GENECONV, Maximum Chi Square (MAXCHI), and Sister Scanning (SISCAN) methods. The results of Simplot analysis showed that the inter-subtype recombinations were both located in the ORF1s, not the ORF2s (Additional file 5: Figure S1). And these recombinants were removed to eliminate the impact of this inter-subtype recombination.

MK test was applied with the coding sequences of all individual proteins among the classic RHDV, RHDVa, RHDVb, and RCV to detect the occurrence of adaptive diversification. The results suggested that viral proteins clearly encountered different evolutionary fates (Additional file 3: Table S3). There were several amino acid changes in the coding sequence of the capsid protein VP60 (nt 1–1740) among the different subtypes. According to Fisher’s exact test of independence, the ratio of these replacement differences to the fixed synonymous was significantly greater than the replacement which happened among intra-subtype versus the synonymous polymorphisms (*P* < 0.01) (Additional file 3: Table S3). Conversely, the results for the coding sequences of the other proteins did not show a similar pattern of evolution. All these results suggested that the accumulation of amino acid changes in VP60 could be a consequence of adaptive diversification during the division of classic RHDV, RHDVa, and RHDVb.

### Functional divergence of amino acid sites among classic RHDV, RHDVa, and RHDVb

It is well known that the adaptive diversification generally happens at few positions because most amino acids in a protein are subject to functional constraints. Moreover, it has been pointed out that adaptive diversification could result in changes in the physicochemical properties of amino acids at critical sites. To advance our understanding of the adaptive evolution among the classic RHDV, RHDVa RHDVb and RCV, we attempted to track the functional diversification of VP60 of individual amino acid sites using the type-II divergence method in DIVERGE 3 [38, 39]. As shown in Additional file 4: Table S4, we identified 50 putative functional divergence-related sites between classic RHDV and RHDVb, 34 putative functional divergence-related sites in RHDVa versus (vs.) RHDVb, 21 putative functional divergence-related sites in classic RHDV vs, RHDVa. 37 putative functional divergence-related sites in RCV vs. RHDVb. Interestingly, there are 33 identical sites between classic RHDV vs. RHDVb and RHDVa vs. RHDVb, but no common site from classic RHDV vs. RHDVa. It has been reported that putative functional divergence-related sites of VP60 may influence the virulence of RHDV, RHDVb, and RCV [34]. The distribution pattern of the identified sites was generally consistent with the results from the MK test above. Overall, these results suggested that adaptive amino acid changes, mainly in the capsid protein VP60, had taken place during the division of the RHDV progenitor into classic RHDV, RHDVa, and RHDVb.

### Analysis of amino acids of RHDV

**Fig. 2:**
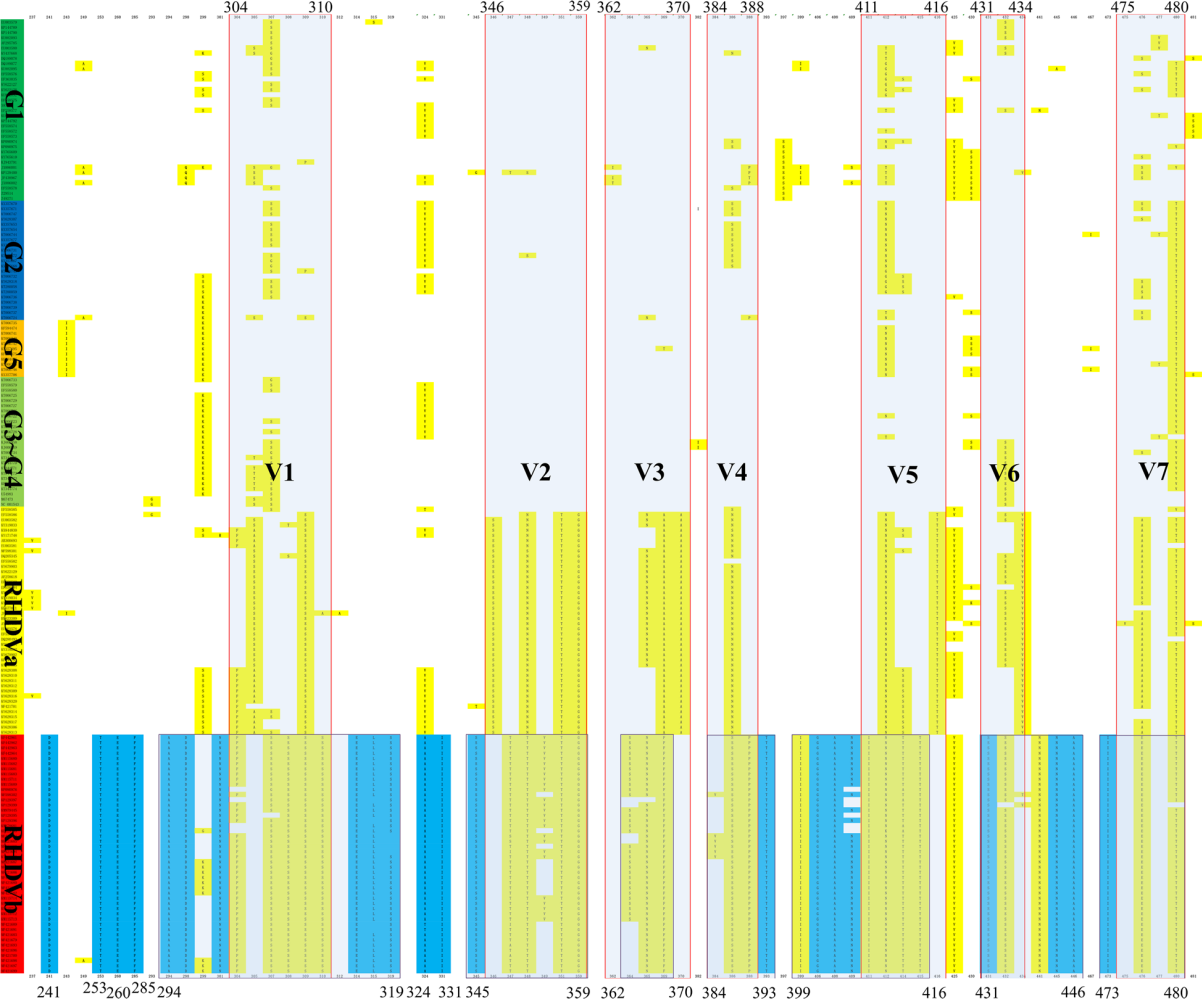
Sequence alignment of VP60 of RHDV isolates. Multiple sequence alignments of VP60 among classic RHDV, RHDVa, and RHDVb isolates. The alignment is shown for the P1 and P2 sub-domain region for residues from 237 to 481. The seven variation regions (V1-V7) that distinguish these isolates are highlighted in gray. The specific variation regions of RHDVb are marked blue

It is well known that there are seven variant regions (V1-V7) in different genetic groups of RHDV isolates. In this study, multiple sequence alignments of the P2 subdomain of VP60 was performed by ClustalW method. We found that not only the range of six variation regions is widening, but also V3 variation region is narrowing in RHDVb. In addition, the amino acids 241, 253, 260, 285, and 331 of RHDVb VP60 were also mutated (Fig. 2).

**Fig. 3:**
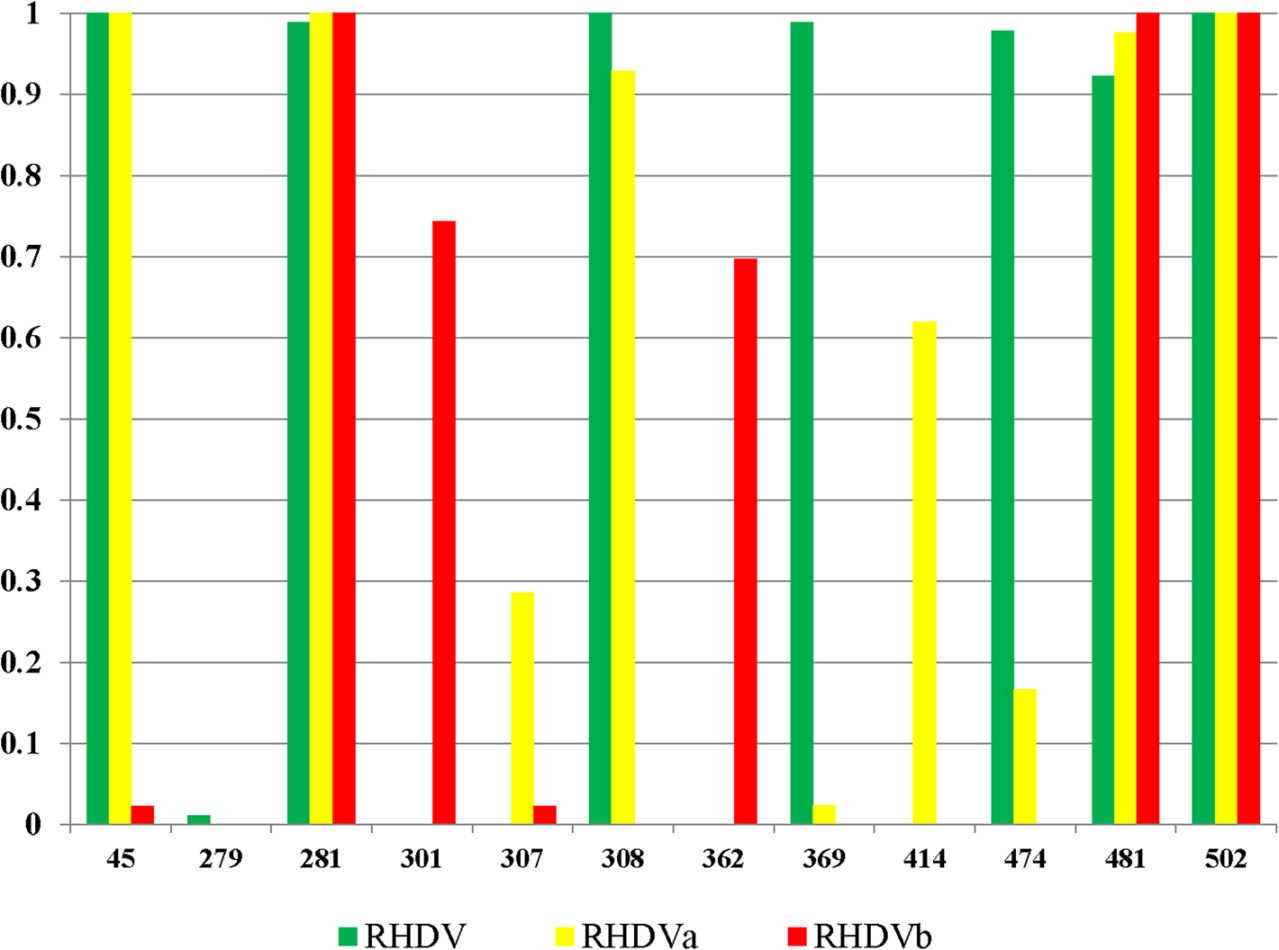
Predicted N-glycosylation sites in VP60 in different subtypes of RHDV. The Y axis is the percentage of potential N-glycosylation of this site in all strains

**Fig. 4:**
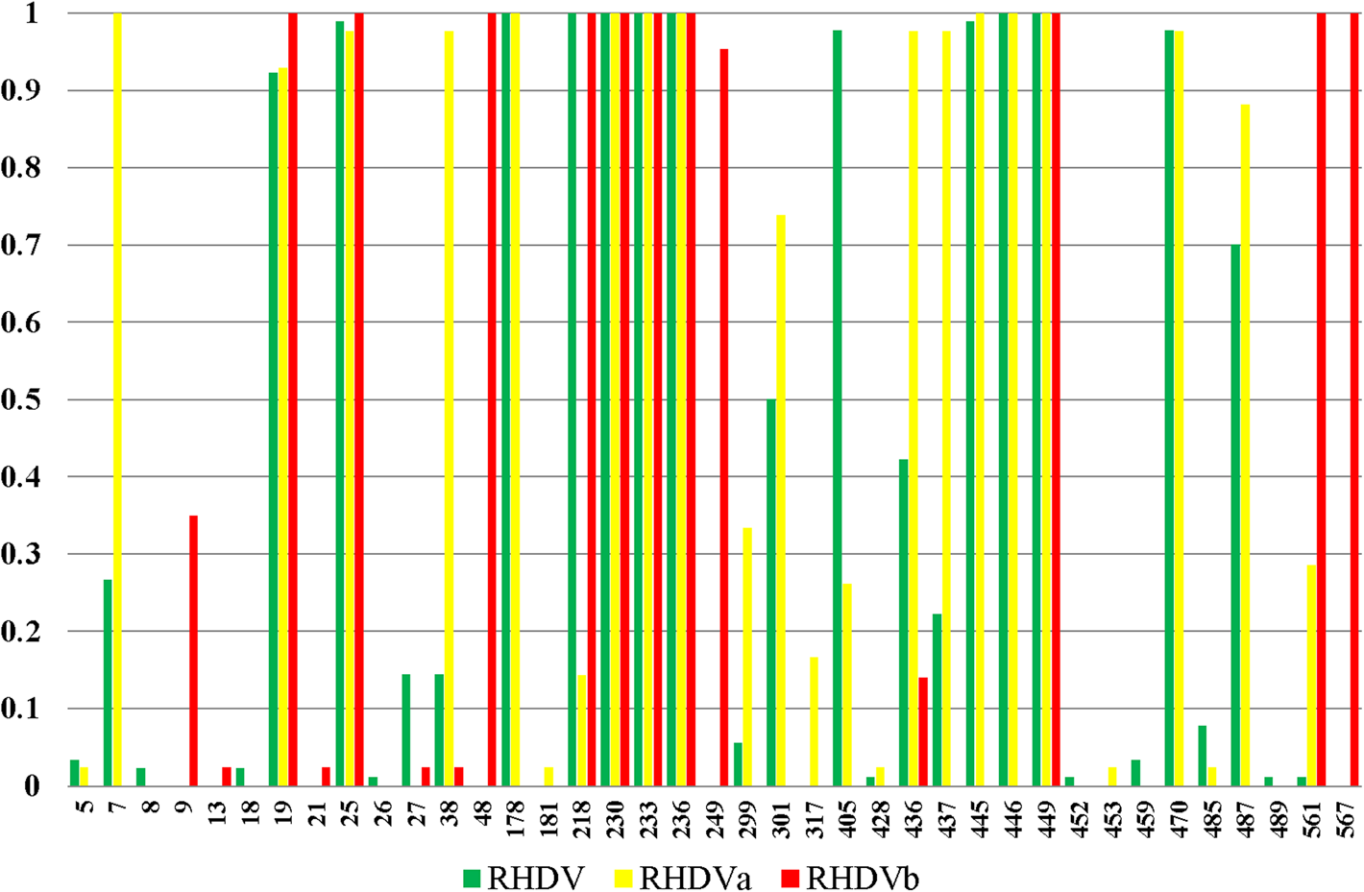
Predicted phosphorylation sites in VP60 in different subtypes of RHDV. The Y axis is the percentage of potential phosphorylation of this site in all strains

N-glycosylation and phosphorylation play an important role in the various biological functions of proteins. It has been reported that they play an important regulatory role in viral infection, replication, and translation [40–42]. We predicted the N-glycosylation and phosphorylation sites of VP60 in the different RHDV subtypes (Fig. 3). VP60 in classic RHDV, RHDVa, and RHDVb included seven, eight, and five N-glycosylation sites, respectively. Interestingly, RHDVa had two specific N-glycosylation sites (aa 307 and 414 in VP60) and lacked one other N-glycosylation site (aa 369 in VP60) compared with classic RHDV. Moreover, RHDVb had two specific N-glycosylation sites (aa 301 and 362 in VP60) and lacked three N-glycosylation sites (aa 45, 308, and 474 in VP60) compared with classic RHDV. The specific N-glycosylation sites in RHDVb may contribute to the difference in host specificity. Phosphorylation site analysis showed that VP60 in classic RHDV, RHDVa, and RHDVb had 19, 21, and 13 phosphorylation sites, respectively (Fig. 4). RHDVa and RHDVb had some phosphorylation sites that differed from those in classic RHDV, which may also contribute to the differences in virulence among classic RHDV, RHDVa, and RHDVb.

## Discussion

VP60 is the major viral structure and immunogenic protein of RHDV [43]. The differences of VP60 among three subtypes of RHDV caused the variances of genetic, antigenic, and epidemiological diversity and immunological response among classic RHDV, RHDVa, and RHDVb. And it’s composed of three domains: the N-terminal arm (NTA, aa 1–65), the shell (S, aa 66–229), a short hinge (aa 230–237) and the protrusion (P, aa 238–579) which is divided into two subdomains (P1 (aa 238–286, 450–466, and 484–579) and P2 (aa 287–449 and 467–483)) [28, 29].

**Fig. 5:**
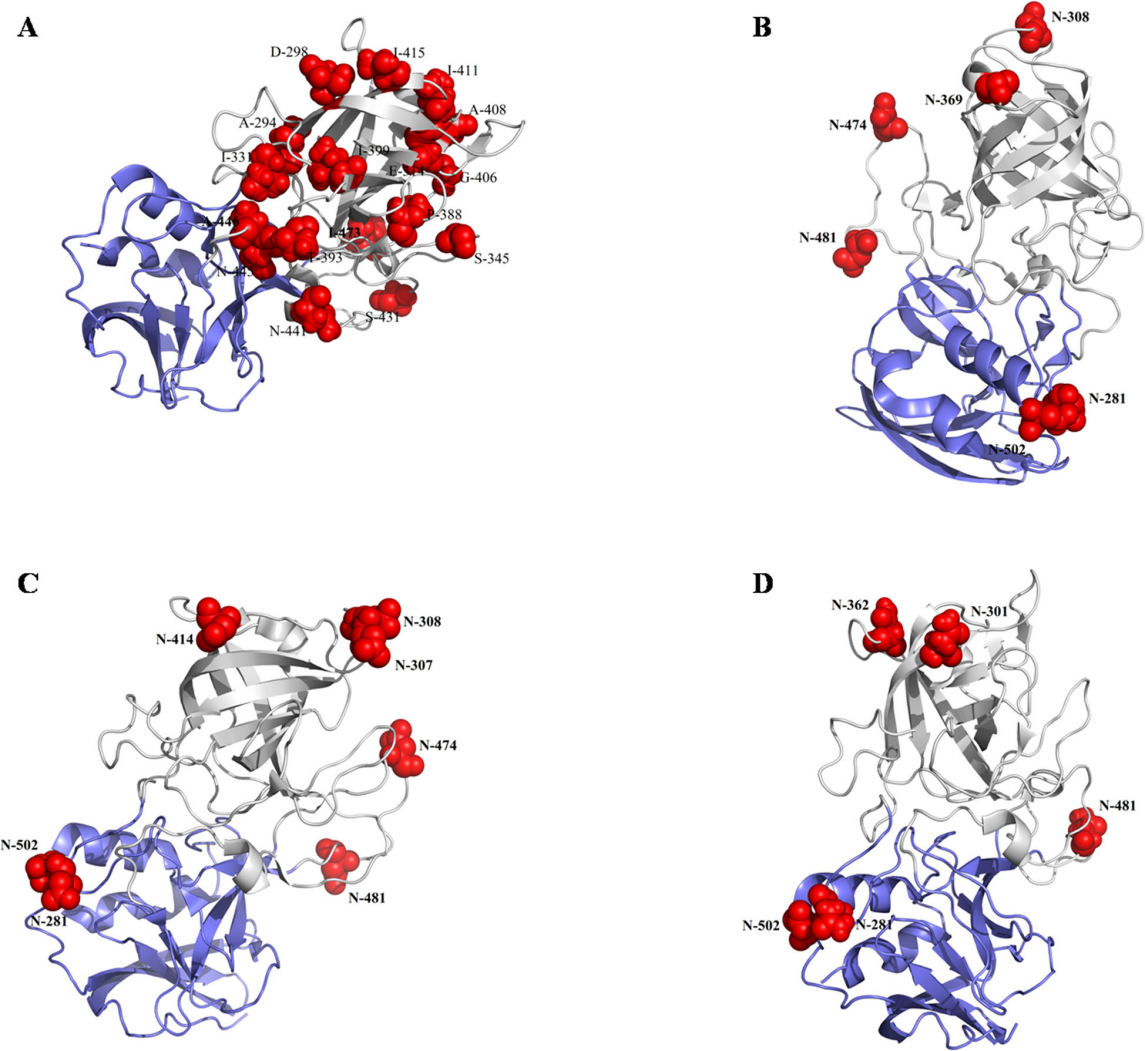
Three-dimensional structure of VP60 P domain. **a** Stick model showing locations of the common divergent sites among classic RHDV, RHDVa, and RHDVb in the VP60 P domain. **b** Stick model showing locations of N-glycosylation sites in classic RHDV VP60 P domain. **c** Stick model showing locations of N-glycosylation sites in the RHDVa VP60 P domain. **d** Stick model showing locations of N-glycosylation sites in RHDVb VP60 P domain

We analyzed the potential functional divergence-related sites in VP60 between RHDVb and RHDV or RHDVa and identified 33 identical sites, including 17 sites in the variant regions of the P2 subdomain, 11 sites in the P1 subdomain, 2 sites in the S domain, and 2 sites in the NTA region (Additional file 4: Table S4). We also found 17 distinct potential functional divergence-related sites in different types of RHDV (Additional file 4: Table S4), including 12 sites in the variant regions of the P2 subdomain, 4 sites in the P1 subdomain, and 1 site in the NTA region. We further analyzed the biological significance of these distinct potential functional divergence-related sites by predicting their precise location in the three-dimensional structure of the VP60 P domain (PDB ID: 4X1W) using PyMOL software (Fig. 5a). Some of the potential functional divergence-related sites in RHDVb were located in a functional domain on the outer surface of RHDV VP60. This domain has been reported to comprise three cavities (1–3), which are responsible for the binding of RHDV to HBGAs. Similar to norovirus structural protein VP1, VP60 of RHDV also has L2 and L6 loops that can bind to HBGA Lewis [32, 44]. We found three potential functional divergence-related sites (aa 331, 345, and 431) of RHDVb VP60 located in the L2 and L6 loops. Other potential functional divergence-related sites may also lead to structural changes in VP60 in RHDVb, potentially allowing RHDVb to bind to new cellular receptors. Further studies are needed to investigate this possibility. However, we speculated that the potential functional divergence-related sites might affect the recognition of HBGAs. Then we analyzed the potential functional divergence-related sites in VP60 between RHDVb and RCV and identified 37 identical sites, including 18 sites in the P2 subdomain, 8 sites in the P1 subdomain, 9 sites in the S domain, and 2 sites in the NTA region (Additional file 4: Table S4) and these sites specially located in the P2 subdomain may play a role in the virulence and organs tropism of RHDV. Although it still needs to be improved. Post-translational modifications of polypeptides or proteins, such as ubiquitination, phosphorylation, and glycosylation, are essential biological features of viral proteins [45]. Glycoproteins make up more than 50% of cellular proteins, and glycosylation is a common post-translational modification in eukaryotes, with roles in important processes such as cell recognition differentiation, development, signal transduction, and the immune response [41, 42]. N-glycosylation site prediction showed that RHDVb had two unique N-glycosylation sites and lacked three N-glycosylation sites in VP60. Glycosylation is known to be related to infection or virulence in many non-enveloped viruses such as rotavirus and hepatitis E virus [46–49]. The positive N-glycosylation sites in VP60 showed that glycosylation might influence the pathogenicity of RHDV [50]. RCV that lacked some of the positive N-glycosylation sites couldn’t infect the rabbits, which also provided evidence for this viewpoint [50]. Deletions of aa 307 and 308 in RHDV VP60 were recently shown to affect the pathogenicity of RHDV [51]. In addition, we further analyzed the biological significance of these distinct potential functional N-glycosylation sites of different subtypes of RHDV by predicting their precise location in the three-dimensional structure of the VP60 P domain (PDB ID: 4X1W) using PyMOL software. As showed in Fig. 5b-d, some of the potential functional N-glycosylation sites in RHDV were located in a functional domain on the outer surface of RHDV VP60. The current results identified aa 307 and 308 as potential N-glycosylation sites in the V1 variable region of VP60. We therefore speculated that the divergence of N-glycosylation sites in VP60 might have affected the virulence of RHDV. Phosphorylation is also an important form of post-translational modification in eukaryotic cells, and plays an important role in the regulation of many biological processes, such as signal transduction, gene expression, and cell division [52, 53]. The current analysis of phosphorylation sites showed that some sites differed among RHDVa, RHDVb, and classic RHDV. However, further studies are needed to determine if these changes might affect the pathogenicity or evolution of RHDV.

## Conclusions

In summary, we employed bioinformatics software to analyze genetic variations among classic RHDV, RHDVa, RHDVb, RCV, and MRCV, covering the near-complete regions of two ORFs, and to predict the N-linked glycosylation and phosphorylation sites in VP60. The genetic relationship between RHDVb and RCV was closer than that with classic RHDV isolates, while RHDVb had two unique N-glycosylation sites but lacked three sites present in classic RHDV and RHDVa VP60. This divergence of N-glycosylation sites might affect the virulence of RHDV. All these results may provide new clues for further investigations of the origin of new types of RHDV and the mechanisms of genetic variation in RHDV.

## Methods

### Sequence data collection and alignment

We selected 184 partial and/or complete genome sequences of RHDV, 46 genome sequences of RCV, and 1 genome sequence of MRCV from GenBank (Additional file 1: Table S1). These sequences were aligned using ClustalW, and the codon reading frames were then checked manually to remove ambiguous codons. The sequence of Z29514 was chosen as the standard sequence.

### Phylogenetic analysis of RHDV isolates

Two phylogenetic trees were constructed based on VP60 [54] and the complete sequence (as aligned to Z29514) using MEGA5.0 software (www.megasoftware.net) with maximum likelihood statistical methods [55]. The confidence levels of the reconstructed trees were evaluated by the bootstrap method with 1000 replicates [56]. RCV and MRCV were chosen as out-groups.

### Recombination detection

Recombination Detection Program (RDP) v4.56 software package was used to detect the recombination of the RHDV genomes [57] with the BOOTSCAN [58], GENECONV [59], Maximum Chi Square (MAXCHI) [60], RDP [61], and Sister Scanning (SISCAN) methods [62]. The *P*-value cutoff was set as 0.05 throughout, and the RHDV genomes happening recombination events were removed.

### Divergence scan

Simplot program was used to assess the divergence of the nucleotide sequences within the three subtypes of RHDV [33]. The reference sequences were chosen randomly as follows: classic RHDV subtype, M67473, and Z49271; RHDVa subtype, DQ205345, and AF258618; and RHDVb subtype, KM979445, and MF421692. A sliding window (200 nt) was moved along the entire coding sequences in steps of 20 nucleotides. The reliability of the recombination sites was assessed by Genetic Algorithm Recombination Detection (GARD, http://www.datamonkey.org).

### McDonald-Kreitman (MK) analysis of coding sequences of individual proteins

McDonald-Kreitman (MK) analysis was used to examine if adaptive evolution had contributed to the diversity among the three subtypes of RHDV by comparing the numbers of synonymous and replacement (non-synonymous) changes, both between and within these phylogenic subtypes [63].. We performed the MK test to identify adaptive diversification in individual protein using http://mkt.uab.es. The divergence was corrected using the Jukes-Cantor model [64], and independence was analyzed by Fisher’s exact test.

### Test for functional divergence in coding sequences of putative diversified proteins

We detected the signature of adaptive diversification at each amino acid sites by implementing the type-II divergence method in DIVERGE 3 covering the ORF1 coding region (nt 10–7044 aligned to Z29514) and ORF2 coding region (nt 7025–7378 aligned to Z29514) of classic RHDV, RHDVa, RHDVb and RCV [38, 39]. The type-II functional divergence at the early stage was calculated after the subtypes split from an ancestor [38, 65]. The result of type-II functional divergence analysis showed that the homologous amino acid residues are conserved in each subtype, but their physicochemical properties are different in each subtype. Moreover, we calculated the coefficient of type II functional divergence (θII) between the individual subtypes [38], and the statistical evaluation was accessed by the Z-score test based on the value of θII and its standard error (θIISE) [39, 65]. The invalid hypothesis is θII = 0, it means that the evolutionary rates are almost identical between the individual subtypes. For a significantly θII value, it means that a valuable change in amino acid physicochemical properties which might have occurred among the subtypes. In addition, in order to predict the amino acid residues of the functional differences, a posterior probability-based confidence measure was introduced with a probability cutoff > 0.95 [38].

### Analysis of amino acids of RHDV

The amino acids in each protein of RHDV were analyzed with MEGA5.0 software (www.megasoftware.net) [55]. The potential N-linked glycosylation and phosphorylation sites of VP60 were also predicted using http://www.cbs.dtu.dk/services/NetNGlyc/ [66] and http://www.dabi.temple.edu/disphos/ [67, 68], respectively.

## Supplementary information

**Additional file 1: Table S1.** RHDV, RCV, and MRCV sequences used in this study.

**Additional file 2: Table S2.** Recombination of RHDV.

**Additional file 3: Table S3.** McDonald-Kreitman analysis of nine individual proteins in classic RHDV, RHDVa, RHDVb, and RCV with complete and partial genome sequences.

**Additional file 4: Table S4.** Estimate of type-II functional divergence among classic RHDV, RHDVa, RHDVb, and RCV subtypes.

**Additional file 5: Figure S1.** Recombination analysis by Simplot. (A) Nucleotide sequence divergence scans of RHDV ORF1. (B) Nucleotide sequence Bootscanning of RHDV ORF1. (C) Nucleotide sequence divergence scans of RHDV ORF2. (D) Nucleotide sequence Bootscanning of RHDV ORF2.

## Data Availability

All genome sequences of RHDV, RCV and MRCV during this study were collected from GenBank (https://www.ncbi.nlm.nih.gov/nuccore/). The GenBank accessions of all sequences during this study are included in this published article and its supplementary information files.

## Abbreviations

EBHSV: European Brown hare Syndrome Virus
MK: McDonald-Kreitman
MRCV: Michigan Rabbit Calicivirus
RCV: Rabbit Calicivirus
RDP: Recombination Detection Program
RHDV: Rabbit Hemorrhagic Disease Virus
