# Supplementary material for: Bioinformatics analysis of capsid protein of different subtypes rabbit hemorrhagic disease virus
Source: BMC Vet Res. 2019 Nov 27;15:423. doi: 10.1186/s12917-019-2161-9 (PMC6882040; doi:10.1186/s12917-019-2161-9)
Supplement: Supplementary file 1 — Additional file 1: Table S1. RHDV, RCV, and MRCV sequences used in this study. [file 12917_2019_2161_MOESM1_ESM.docx]

| RHDV、RCV and MRCV sequences used for this study | | |
| --- | --- | --- |
| GenBank  accession number | Date of collection | Country |
| **RHDV** |  |  |
| Complete genome |  |  |
| AB300693 | 2002 | Japan |
| AF258618 | 2000 | USA |
| AY523410 |  | China |
| DQ189077 |  | Bahrain |
| DQ205345 | 1997 | China |
| DQ280493 |  | China |
| EF363035 |  | United Kingdom |
| EF558572 | 1996 | Germany |
| EF558573 |  | Germany |
| EF558574 |  | Germany |
| EF558575 |  | United Kingdom |
| EF558576 |  | Germany |
| EF558577 |  | Germany |
| EF558578 |  | Germany |
| EF558579 |  | New Zealand |
| EF558580 |  | New Zealand |
| EF558581 |  | Germany |
| EF558582 |  | Germany |
| EF558583 |  | Germany |
| EF558584 |  | Germany |
| EF558586 |  | Germany |
| EU003578 | USA | USA |
| EU003579 | 1990 | Italy |
| EU003581 | 2001 | USA |
| EU003582 | 2001 | USA |
| HM623309 | 2009 | China |
| JF412629 | 2005 | China |
| JX886001 | 2006 | Portugal |
| JX886002 | 1995 | Portugal |
| KF677011 | 2012 | Poland |
| KJ943791 | 1996 | Portugal |
| KM878681 | 2011 | Spain |
| KM979445 | 2012 | Portugal |
| KP144789 | 1989 | Poland |
| KP144790 | 1988 | Poland |
| KP144791 | 2004 | Poland |
| KP144792 | 1994 | Poland |
| KU882092 | 1998 | Poland |
| KU882093 | 1994 | Poland |
| KU882094 | 2004 | Poland |
| KU882095 | 2000 | Poland |
| KX844830 | 2004 | China |
| KY171748 | 2007 | China |
| KY319031 | 2015 | Poland |
| KY319032 | 2013 | Poland |
| KY319033 | 2004 | Poland |
| KY319034 | 2013 | Poland |
| KY319035 | 2005 | Poland |
| KY437668 | 2016 | China |
| KY679902 | 2004 | Poland |
| KY679903 | 2013 | Poland |
| KY679904 | 2013 | Poland |
| KY679905 | 2014 | Poland |
| M67473 |  |  |
| NC_001543 |  |  |
| U54983 |  | Czekh |
| X87607 |  |  |
| Z29514 |  |  |
| Z49271 |  | Spain |
| Partial CDS |  |  |
| AF295785 | 1989 | Mexico |
| DQ189078 |  | Saudi Arabia |
| EF558585 |  | Germany |
| EU003580 | 1990 | South Korea |
| JF438967 | 1997 | Portugal |
| KF442961 | 2013 | Portugal |
| KF442962 | 2013 | Portugal |
| KF442963 | 2013 | Portugal |
| KF442964 | 2013 | Portugal |
| KF594473 | 1991 | Australia |
| KF594474 | 2006 | Australia |
| KF594475 | 2007 | Australia |
| KF594476 | 2009 | Australia |
| KJ606958 | 1998 | Australia |
| KJ606959 | 1998 | Australia |
| KM115680 | 2013 | Portugal |
| KM115681 | 2014 | Portugal |
| KM115682 |  | Portugal |
| KM115683 |  | Portugal |
| KM115689 | 2014 | Portugal |
| KM115711 | 2013 | Portugal |
| KM115712 | 2014 | Portugal |
| KM115713 | 2014 | Portugal |
| KM115714 |  | Portugal |
| KM115715 |  | Portugal |
| KM115716 |  | Portugal |
| KP090974 | 1994 | Portugal |
| KP090975 | 1997 | Portugal |
| KP090976 | 2012 | Spain |
| KP129395 |  | Spain |
| KP129396 |  | Spain |
| KP129397 |  | Spain |
| KP129398 | 2010 | Spain |
| KP129399 |  | Spain |
| KP129400 | 2011 | Spain |
| KT006721 | 2013 | New Zealand |
| KT006722 | 2012 | New Zealand |
| KT006723 | 2012 | New Zealand |
| KT006724 | 2013 | New Zealand |
| KT006725 | 2013 | New Zealand |
| KT006726 | 2014 | New Zealand |
| KT006727 | 2014 | New Zealand |
| KT006728 | 2014 | New Zealand |
| KT006729 | 2014 | New Zealand |
| KT006730 | 2013 | New Zealand |
| KT006731 | 2007 | Australia |
| KT006732 | 2014 | Australia |
| KT006733 | 1999 | Australia |
| KT006734 | 1999 | Australia |
| KT006735 | 2000 | Australia |
| KT006736 | 2000 | Australia |
| KT006737 | 2002 | Australia |
| KT006738 | 2003 | Australia |
| KT006739 | 2004 | Australia |
| KT006740 | 2004 | Australia |
| KT006741 | 2005 | Australia |
| KT006742 | 2006 | Australia |
| KT006743 | 2007 | Australia |
| KT006744 | 2008 | Australia |
| KT006745 | 2009 | Australia |
| KT006746 | 2012 | Australia |
| KT006747 | 2013 | Australia |
| KT280058 | 2015 | Australia |
| KT280059 | 2015 | Australia |
| KT280060 | 2015 | Australia |
| KT344770 |  | Australia |
| KT344771 |  | Australia |
| KT344772 |  | Australia |
| KT344773 |  | Australia |
| KT344774 |  | Australia |
| KT344775 |  | New Zealand |
| KX357653 | 2009 | Australia |
| KX357654 | 2009 | Australia |
| KX357667 | 2009 | Australia |
| KX357668 | 2009 | Australia |
| KX357670 | 2010 | Australia |
| KX357671 | 2010 | Australia |
| KX357672 | 2009 | Australia |
| KX357677 | 2007 | Australia |
| KX357678 | 2007 | Australia |
| KX357695 | 2008 | Australia |
| KX357706 | 2013 | Australia |
| KY622127 | 1998 | Portugal |
| KY622128 | 1998 | Portugal |
| KY622129 | 1999 | Portugal |
| KY628306 | 2014 | Australia |
| KY628307 | 2013 | Australia |
| KY628308 | 2014 | Australia |
| KY628309 | 2013 | Australia |
| KY628310 | 2014 | Australia |
| KY628311 | 2014 | Australia |
| KY628312 | 2014 | Australia |
| KY628313 | 2014 | Australia |
| KY628314 | 2014 | Australia |
| KY628315 | 2014 | Australia |
| KY628316 | 2014 | Australia |
| KY628317 | 2015 | Australia |
| KY628318 | 2014 | Australia |
| KY628320 | 2015 | Australia |
| KY765609 | 1994 | Portugal |
| KY765610 | 1994 | Portugal |
| MF421679 | 2015 | Australia |
| MF421680 | 2016 | Australia |
| MF421681 | 2016 | Australia |
| MF421682 | 2016 | Australia |
| MF421683 | 2016 | Australia |
| MF421684 | 2016 | Australia |
| MF421685 | 2016 | Australia |
| MF421686 | 2016 | Australia |
| MF421687 | 2016 | Australia |
| MF421688 | 2016 | Australia |
| MF421689 | 2016 | Australia |
| MF421690 | 2016 | Australia |
| MF421691 | 2016 | Australia |
| MF421692 | 2016 | Australia |
| MF421693 | 2016 | Australia |
| MF421694 | 2016 | Australia |
| MF421696 | 2016 | Australia |
| MF421697 | 2016 | Australia |
| MF421698 | 2016 | Australia |
| MF421699 | 2016 | Australia |
| MF421700 | 2016 | Australia |
| MF421701 | 2015 | Australia |
| MF598301 | 2008 | South Korea |
| MF598302 | 2016 | Australia |
| **RCV** |  |  |
| Complete genome |  |  |
| EU871528 | 2007 | Australia |
| NC_011704 | 2007 | Australia |
| Partial CDS |  |  |
| KX357655 | 2013 | Australia |
| KX357656 | 2014 | Australia |
| KX357657 | 2014 | Australia |
| KX357658 | 2012 | Australia |
| KX357659 | 2008 | Australia |
| KX357660 | 2010 | Australia |
| KX357661 | 2011 | Australia |
| KX357662 | 2011 | Australia |
| KX357663 | 2012 | Australia |
| KX357664 | 2014 | Australia |
| KX357665 | 2007 | Australia |
| KX357666 | 2008 | Australia |
| KX357669 | 2010 | Australia |
| KX357673 | 2007 | Australia |
| KX357674 | 2007 | Australia |
| KX357675 | 2007 | Australia |
| KX357676 | 2010 | Australia |
| KX357679 | 2007 | Australia |
| KX357680 | 2007 | Australia |
| KX357681 | 2011 | Australia |
| KX357682 | 2007 | Australia |
| KX357683 | 2007 | Australia |
| KX357684 | 2007 | Australia |
| KX357685 | 2007 | Australia |
| KX357686 | 2007 | Australia |
| KX357687 | 2007 | Australia |
| KX357688 | 2007 | Australia |
| KX357689 | 2007 | Australia |
| KX357690 | 2007 | Australia |
| KX357691 | 2007 | Australia |
| KX357692 | 2007 | Australia |
| KX357693 | 2007 | Australia |
| KX357694 | 2009 | Australia |
| KX357696 | 2009 | Australia |
| KX357697 | 2010 | Australia |
| KX357698 | 2010 | Australia |
| KX357699 | 2009 | Australia |
| KX357700 | 2010 | Australia |
| KX357701 | 2007 | Australia |
| KX357702 | 2009 | Australia |
| KX357703 | 2009 | Australia |
| KX357704 | 2009 | Australia |
| KX357705 | 2009 | Australia |
| KX357707 | 2013 | New Zealand |
| **MRCV** |  |  |
| GQ166866 | 2001 | USA |
