# Supplementary material for: Bioinformatics analysis of capsid protein of different subtypes rabbit hemorrhagic disease virus
Source: BMC Vet Res. 2019 Nov 27;15:423. doi: 10.1186/s12917-019-2161-9 (PMC6882040; doi:10.1186/s12917-019-2161-9)
Supplement: Supplementary file 2 — Additional file 2: Table S2. Recombination of RHDV. [file 12917_2019_2161_MOESM2_ESM.docx]

**Table. S2 The recombination of RHDV**

| Recomb. | Major  parent | Minor  parent | Detection methods | | | | | | |
| --- | --- | --- | --- | --- | --- | --- | --- | --- | --- |
|  |  |  | R | G | B | M | C | S | T |
| MF598302 | KY628314 | KT280060 | + | + | + | + | + | + | + |
| MF421679 | JX886001 | KF442964 | + | + | + | + | + | + | + |
| KP129396 | KJ606959 | KF442964 | + | + | + | + | + | + | + |
| KY628317 | KM115683 | KY171748 | + | + | + | + | + | + | + |
| KY765609 | Unknown | Z49271 | + | + | + | + | + | + | + |
| KY628312 | KM115682 | AB300693 | + | + | + | + | + | + | + |
| EF558585 | AF258618 | X87607 | + | + | + | + | + | + | + |
| EF558586 | EF558583 | EF558580 | + | + | + | - | - | - | - |
