# Supplementary material for: Bioinformatics analysis of capsid protein of different subtypes rabbit hemorrhagic disease virus
Source: BMC Vet Res. 2019 Nov 27;15:423. doi: 10.1186/s12917-019-2161-9 (PMC6882040; doi:10.1186/s12917-019-2161-9)
Supplement: Supplementary file 3 — Additional file 3: Table S3. McDonald-Kreitman analysis of nine individual proteins in classic RHDV, RHDVa, RHDVb, and RCV with complete and partial genome sequences. [file 12917_2019_2161_MOESM3_ESM.docx]

**Table. S3 McDonald-Kreitman test on nine individual proteins of classic RHDV, RHDVa, and RHDVb with the complete and partial genome sequences.**

|  | Classic RHDV vs.  RHDVb | | RHDVa vs. RHDVb | | RCV vs. RHDVb | |
| --- | --- | --- | --- | --- | --- | --- |
|  | Fixed | Polymorphism | Fixed | Polymorphism | Fixed | Polymorphism |
| p16 |  |  |  |  |  |  |
| Replacement | 0.00 | 137 | 0.00 | 111 | 0.00 | 119 |
| Synonymous | 1.00 | 210 | 1.00 | 172 | 1.00 | 177 |
| p-value | 0.417 |  | 0.420 |  | 0.410 |  |
| p23 |  |  |  |  |  |  |
| Replacement | 0.00 | 144 | 0.00 | 143 | 0.00 | 139 |
| Synonymous | 1.00 | 447 | 3.04 | 421 | 1.00 | 480 |
| p-value | 0.591 |  | 0.309 |  | 0.589 |  |
| helicase |  |  |  |  |  |  |
| Replacement | 0.00 | 123 | 0.00 | 130 | 1.00 | 96 |
| Synonymous | 1.00 | 742 | 3.02 | 751 | 1.00 | 833 |
| p-value | 0.683 |  | 0.469 |  | 0.066 |  |
| p29 |  |  |  |  |  |  |
| Replacement | 0.00 | 112 | 0.00 | 115 | 1.00 | 126 |
| Synonymous | 1.00 | 551 | 3.03 | 521 | 3.03 | 623 |
| p-value | 0.651 |  | 0.413 |  | 0.668 |  |
| Vpg |  |  |  |  |  |  |
| Replacement | 0.00 | 40 | 0.00 | 46 | 0.00 | 30 |
| Synonymous | 1.00 | 215 | 1.00 | 223 | 1.00 | 225 |
| p-value | 0.664 |  | 0.648 |  | 0.713 |  |
| protease |  |  |  |  |  |  |
| Replacement | 0.00 | 44 | 0.00 | 40 | 0.00 | 34 |
| Synonymous | 1.00 | 324 | 1.00 | 327 | 1.00 | 319 |
| p-value | 0.711 |  | 0.725 |  | 0.743 |  |
| RdRp |  |  |  |  |  |  |
| Replacement | 0.00 | 210 | 0.00 | 356 | 0.00 |  |
| Synonymous | 1.00 | 1139 | 1.00 | 1035 | 5.04 | 336 |
| p-value | 0.576 |  | 0.557 |  | 0.201 | 1039 |
| VP60 |  |  |  |  |  |  |
| Replacement | 36.70 | 149 | 45.03 | 107 | 53.45 | 128 |
| Synonymous | 38.13 | 793 | 92.82 | 564 | 64.97 | 753 |
| p-value | <0.01 |  | <0.01 |  | <0.01 |  |
| VP10 |  |  |  |  |  |  |
| Replacement | 1.00 | 23 | 10.25 | 33 | 1.00 | 63 |
| Synonymous | 3.18 | 64 | 13.50 | 95 | 5.24 | 111 |
| p-value | 0.911 |  | 0.085 |  | 0.300 |  |

The significance according to the Fisher's exact test (two tailed).
