# Supplementary material for: Bioinformatics analysis of capsid protein of different subtypes rabbit hemorrhagic disease virus
Source: BMC Vet Res. 2019 Nov 27;15:423. doi: 10.1186/s12917-019-2161-9 (PMC6882040; doi:10.1186/s12917-019-2161-9)
Supplement: Supplementary file 4 — Additional file 4: Table S4. Estimate of type-II functional divergence among classic RHDV, RHDVa, RHDVb, and RCV subtypes. [file 12917_2019_2161_MOESM4_ESM.docx]

Table. S4 The estimate of the type-II functional divergence among the VP60 of classic RHDV, RHDVa, RHDVb and RCV

|  | Estimate  (Z-score) | Divergence-related sites^b^  (at P_II_ >0.95) |
| --- | --- | --- |
| classic RHDV  vs.  RHDVb | θ_II_^a^ =0.047 ±0.012  (14.35,P<0.01,Z-score test) | 13,43,46,48,88,183,241,253,260,285,294,298,301,304,308,309,310,314,315,319,331,345,348,351,359,365,369,388,393,399,406,408,411,415,431,441,445,446,,455,473,487,496,518,526,548,551,553,562,567,573(in VP60) |
| RHDVa  vs.  RHDVb | θ_II_^a^ =0.029 ±0.008  (14.23,P<0.01,Z-score test) | 7,43,46,48,88,183,241,253,260,285,294,298,314,331,345,388,393,399,406,408,411,415,431,441,445,446,473,487,518,526,551,553,562,567(in VP60) |
| classic RHDV vs.  RHDVa | θ_II_^a^ =0.029 ±0.012  (17.57,P<0.01,Z-score test) | 37,50,27,219,305,309,346,348,351,359,365,369,370,386,412,416,432,434,476,480,473(in VP60) |
| RCV  vs.  RHDVb | θ_II_^a^ =0.027 ±0.011  (9.85,P<0.01,Z-score test) | 8,12,93,104,135,137,145,183,205,207,226,241,260,293,294,298,301,309,322,335,352,359,371,386,388,411,421,431,432,434,457,459,476,487,520,541,572(in VP60) |

^a^ Coefficient of type-II functional divergence.

^b^ The numbers of amino acid sites represent their position as aligned to Z29514.
