# Supplementary figures and images for: Bioinformatics analysis of capsid protein of different subtypes rabbit hemorrhagic disease virus
Source: BMC Vet Res. 2019 Nov 27;15:423. doi: 10.1186/s12917-019-2161-9 (PMC6882040; doi:10.1186/s12917-019-2161-9)

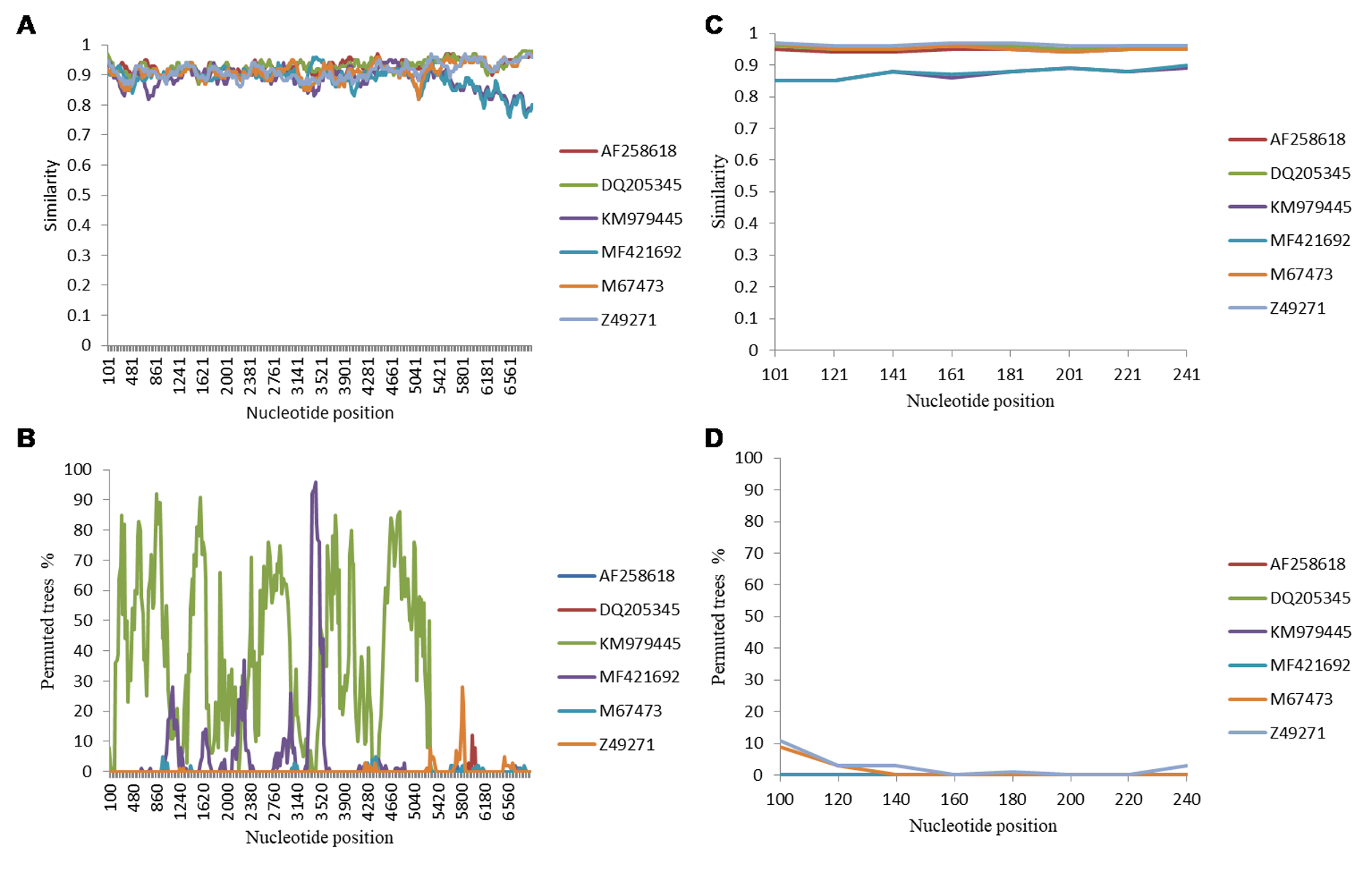

Supplement: Supplementary file 5 — Additional file 5: Figure S1. Recombination analysis by Simplot. (A) Nucleotide sequence divergence scans of RHDV ORF1. (B) Nucleotide sequence Bootscanning of RHDV ORF1. (C) Nucleotide sequence divergence scans of RHDV ORF2. (D) Nucleotide sequence Bootscanning of RHDV ORF2. [file 12917_2019_2161_MOESM5_ESM.tif]
